# Integrated Multitrophic Aquaculture By-Products with Added Value: The Polychaete *Sabella spallanzanii* and the Seaweed *Chaetomorpha linum* as Potential Dietary Ingredients

**DOI:** 10.3390/md17120677

**Published:** 2019-11-30

**Authors:** Loredana Stabili, Ester Cecere, Margherita Licciano, Antonella Petrocelli, Benedetto Sicuro, Adriana Giangrande

**Affiliations:** 1 Institute of Water Research (IRSA) C.N.R, 74123 Taranto, Italy; ester.cecere@irsa.cnr.it (E.C.); antonella.petrocelli@irsa.cnr.it (A.P.); 2 Department of Biological and Environmental Sciences and Technologies, University of Salento, Via Provinciale Lecce-Monteroni, 73100 Lecce, Italy; margherita.licciano@unisalento.it (M.L.); adriana.giangrande@unisalento.it (A.G.); 3 Department of Veterinary Science, University of Turin, L.go Braccini 2, 10095 Grugliasco (Torino), Italy; benedetto.sicuro@unito.it

**Keywords:** *Chaetomorpha linum*, *Dicentrarchus labrax*, fish growth, fish nutrition, innovative meal, *Sabella spallanzanii*

## Abstract

Aquaculture expansion is limited by the negative environmental impact of the waste and the need for alternative sources in the diet of reared fish. In this framework, for the first time, the survival rates, biomass gain, and fatty acid profiles of the polychaete *Sabella spallanzanii* and the macroalga *Chaetomorpha linum*, reared/cultivated as bioremediators in an integrated multitrophic aquaculture system (IMTA), were evaluated for their potential reuse applications. Results showed that these organisms represent a natural source of omega-3 and omega-6. On account of the overall results and the high biomass obtained as by-products, a preliminary study was performed employing both *S. spallanzanii* and *C. linum* as new dietary ingredients to feed different sized *Dicentrarchus labrax*. Fish survival rate, biomass growth, and specific growth rate were determined resulting in no significant differences between control and treated fishes. Histological analyses showed no alterations of the stomach tunica mucosa and submucosa in treated fishes. The eco-friendly approaches applied in the here-realized IMTA system could guarantee the achievement of sustainable by-products represented by the bioremediators *S. spallanzanii* and *C. linum,* as well as their reliability as a natural source of compounds beneficial to fish and human health.

## 1. Introduction

Aquaculture currently provides almost 45% of the world’s fisheries products and an increased production up to almost 62% is expected by 2030 [1]. However, its expansion is limited by several factors including the need to develop new alternative diets for reared fish and the reduction of the impact of this activity on the marine environment. In this context, in recent years, a substantial proportion of the research has been aimed at creating integrated multi-trophic aquaculture (IMTA) systems. Here, the simultaneous rearing of fed species with bioremediators, which can use the nutrient surplus for their growth, either inorganic (e.g., seaweeds or other aquatic vegetation) or organic (e.g., deposit- and suspension-feeders), can allow the attainment of a sustainable aquaculture [2]. Indeed, IMTA has the potential to produce economically exploitable biomasses and provide biomitigative services at the same time, which can be beneficial for both ecosystem and human health. In the light of a more sustainable aquaculture industry, several studies have shown that microbial contamination within farms is reduced by some filter-feeders bioremediators (e.g., oysters, mussels, clams, polychaetes, and sponges) able to process large volumes of waters for their food requirements, efficiently retaining small particles including bacteria [3,4,5,6,7,8,9]. Bioremediation is also accomplished by macroalgae used to reduce the nitrogen load, especially in ammoniacal form, produced by fish metabolism and by the processes of decomposition of uneaten feed. Algae are commonly used in co-culture with bivalves [10] although numerous variations to this basic scheme have been tested. The farming of fish with bioremediators allows the conversion of the uneaten feed, wastes, and nutrients into biomass that can be removed and potentially managed as a valuable by-product. Indeed, marine biomass has an enormous potential as a source for nutritional, therapeutic, and functional ingredients, which may be used to make products for animal and human consumption [11]. At present, the interest of the food industry related to aquaculture activity is mainly focused on functional foods, consisting of one or several functional ingredients of natural origin able to further supply fish and human health benefit [12,13]. The considerable content of high-quality proteins with all the essential amino acids makes fishmeal a very good ingredient in feeds and not substitutable with crop plants, in which proteins, conversely, lack most of these amino acids, such as lysine, methionine, threonine, and tryptophan [14]. Fishmeal is also rich in lipids with high-quality polyunsaturated n-3 and n-6 fatty acids (PUFAs), which are considered beneficial to human cardiovascular health. Although marine fish represents the main source of eicosapentaenoic acid (EPA) and docosahexaenoic acid (DHA) in the formulation of fish feeds, there is an urgent need for an alternative and sustainable source of n-3 long chain PUFAs on account of the depletion of wild fish stocks and the pollution of the marine environment. Recently, Stabili et al. [15] suggested that the very common Mediterranean polychaete *Sabella spallanzanii,* obtained as by-product of bioremediation in aquaculture farms, could be employed as a dietary supplement for fish nourishment on account of the high protein content as well as the presence of certain amino acids that could improve palatability of the worms when included in fish feeds [14,15]. Indeed, *S. spallanzanii* shows an interesting amino acids profile including lysine, methionine, and threonine in an amount comparable with fish meal, other than some amino acids present in excess such as glycine, arginine, cysteine, histidine, and glutamic acid. 

In this framework, in the present paper the rearing/cultivation of the two bioremediators, *S. spallanzanii* and the macroalga *Chaetomorpha linum*, in an integrated rearing fish system was realized, for the first time, in an integrated multitrophic aquaculture system (IMTA) in the Mediterranean Sea. The survival rates, biomass gain, and fatty acid profiles of the two bioremediators were evaluated for their potential reuse applications, including their employment as ingredients of an innovative fish feed for preliminary assays on the European sea bass *Dicentrarchus labrax* juveniles. Polychaete worms, in particular nereids commonly known as omega worms, have been already used in aquaculture as feed [16,17,18,19]. Additionally, macroalgae have been widely tested as dietary components in aquaculture plants and recognized as an alternative source of fatty acids [20,21,22,23]. Histological analyses on the sea bass fish juveniles were also performed in order to obtain a first insight on the potential stomach distress and damage of the epithelium due to the innovative feed. 

## 2. Results

### 2.1. Rearing/Cultivation of Bioremediators in IMTA

**Figure 1:**
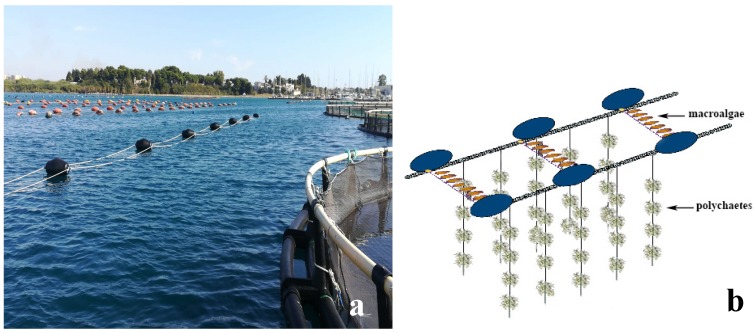
Rearing/cultivation of bioremediators in the IMTA system: (**a**) Fish cages of the integrated multi-trophic aquaculture (IMTA) system in the Mar Grande of Taranto and long-line system; (**b**) detail of the location of the bioremediators. As indicated by the arrows, the algae were horizontally arranged at 1 m depth within a typical long-line system, while polychaetes were placed vertically in polypropylene nets and placed around the fish cages.

The monthly monitoring of the well-being and growth of seaweeds placed in the realized IMTA system (Figure 1) showed, during the six months of *C. linum* cultivation, interesting cultivation performances. 

**Figure 2:**
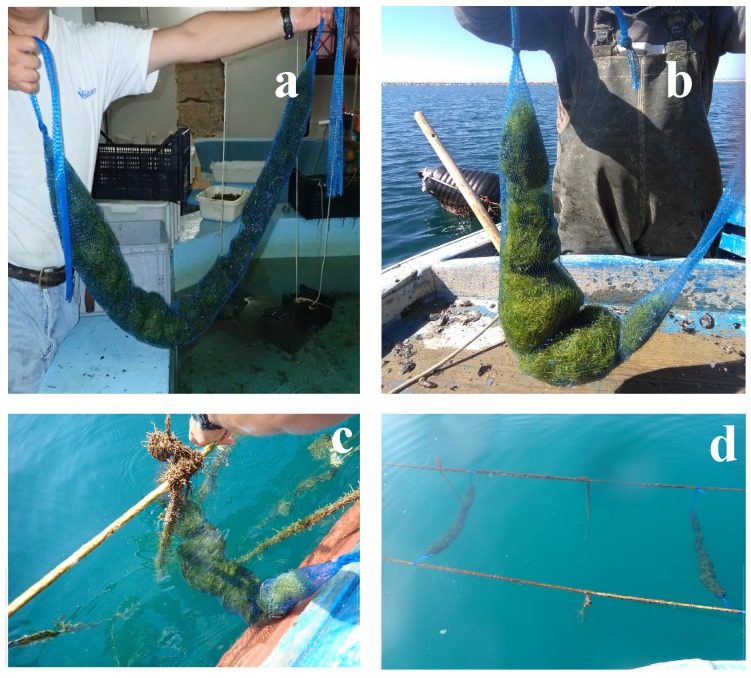
Cultivation trials of *Chaetomorpha linum* in the realized IMTA system: (**a**,**b**) Algae arranged in cultivation sockets; (**c**) *C. linum* located in the farm; (**d**) algae in net sacks hung at 1 m depth within a typical long-line system.

High survival values and significant increases in biomass produced in short time intervals were indeed recorded. In particular, for *C. linum* a maximum specific growth rate (SGR) equal to 5% was calculated in a six months trial (Figure 2). 

**Figure 3:**
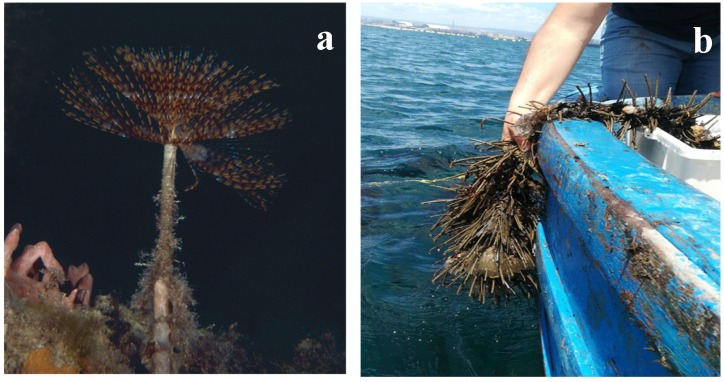
Rearing trials of the polychaete *Sabella spallanzanii* in the integrated multi-trophic aquaculture (IMTA) system: (**a**) Specimen of *S. spallanzanii;* (**b**) polychaetes arranged in polypropylene nets, which were hung vertically within a typical long-line system.

In the IMTA system, about 1428 specimens of *S. spallanzanii* were estimated in each collector for a total of 360,000 individuals in the whole system, with an initial biomass of 0.2 t. After the six months of permanence, the polychaete biomass was of 0.645 t (Figure 3). 

Within the first three months of rearing, a biomass increase of about 79.3% was recorded, while in the last three months the biomass mean value increased with a further gain of 20.7%. As far as the survivorship of *S. spallanzanii* in the farming plant, worms showed a very low mortality rate during all months of observations. In particular, after the six months of rearing, a mortality rate of about 15% was recorded. 

### 2.2. Total Lipid and Fatty Acid Composition

The total mean lipid content of *C. linum* corresponded to 9.4 ± 2.4 mg/g dry weight (DW). In the case of *S. spallanzanii*, the mean lipid content was 80 ± 4.2 mg/g DW.

**Figure 4:**
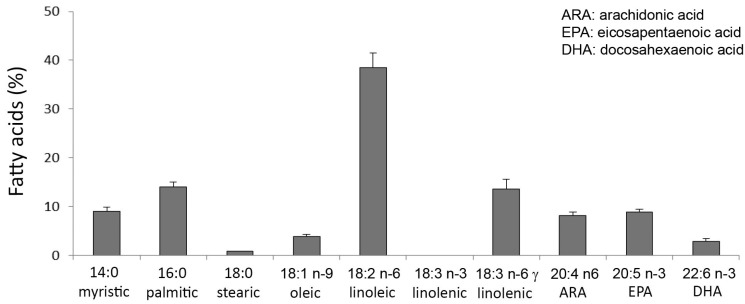
Fatty acid composition of *Chaetomorpha linum*.

The fatty acid profile of total lipids extracted from *C. linum* is shown in Figure 4. Polyunsaturated fatty acids (PUFAs) were the most abundant, accounting for 71.97% of total FAs, and the most abundant PUFAs were linoleic acid (18:2 n-6), 18:3 n-6 γ linolenic acid, the n-3 eicosapentaenoic acid (EPA, 20:5 n-3), and the n-6 arachidonic acid (ARA, 20:4 n-6), and accounting for 38.46%, 14%, 8.83%, and 8.14% of total FAs, respectively (Figure 4a). The n-3 docosahexaenoic acid (DHA, 22:6 n-3) represented 2.91%. Saturated fatty acids (SFAs) represented 23.83% of total fatty acids (FAs). Palmitic acid (16:0) was the prevalent SFA (14.03% of total FAs), followed by myristic acid (14:0; 9% of total FAs). Monounsaturated fatty acids (MUFAs) showed the lowest percentage (4.2% of total FAs) and among them oleic acid (18:1 n-9) prevailed. The ratio of n-6:n-3 fatty acids was about 5. 

The fatty acid profile of *S. spallanzanii* is shown in Table 1. Palmitic acid (16:0) was the predominant SFA (accounting for 26.18% of total lipids) followed by myristic acid (14:0) and stearic acid (18:0). Palmitoleic acid (16:1) prevailed among MUFAs and 16-docosadienoic acid (22:2, n-6) was the most abundant PUFA. The ratio n -6/n-3 fatty acids was about 1.7. 

### 2.3. Feed Formulation and Preliminary Fish Growth Trials 

The prepared fish diets are reported in Table 2 and as shown in Table 3, both the control (CTRL) and innovative (IM) feeds were isoenergetic, isoproteic, and isolipidic. During both the preliminary fish growth trials (May–September), in which the experimental feeds were employed, the mean temperature ranged between 20.6 and 23 °C and dissolved oxygen between 6.2 and 7.4 mg/L. All the measured water parameters resulted in the physiological range for *D. labrax*. In both the experimental trials, differences on the biomass gain, specific growth rate, and survival rate between the control and the treatment were not statistically significant (Table 4). 

In particular, when the innovative meal was employed, biomass gain reached a value of 0.55 ± 0.01 g in the first trial and 2.10 ± 0.12 g in the second trial. These values did not differ statistically from those recorded for the control feed accounting 0.62 ± 0.04 g in the first trail and 2.87 ± 0.15 g in the second trial. As regards the specific growth rate, the value recorded for IM group in the first trial was 4.14% ± 0.16% and 2.4% ± 0.11% in the second trial. 

These values were not statistically different from those recorded for the respective controls in both the trials. A negligible fish mortality was recorded for both the feeds at the end of each trial and in particular the survival rates were 87% ± 2% for CTRL and 96% ± 2% for IM with the lowest size of fish (first trial), and 85% ± 3% for CTRL and 94% ± 2% for IM in the second trial. 

### 2.4. Fish Histological Analyses

**Figure 5:**
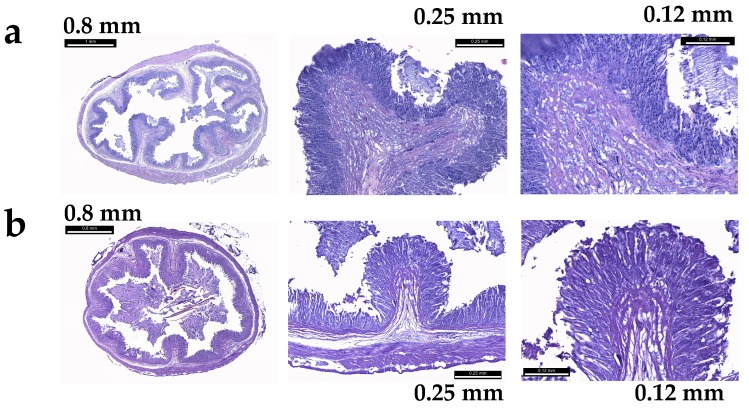
Stomach histological analyses of the fish feed with the experimental diets: (**a**) Fish fed with the control meal; (**b**) fish fed with the innovative meal.

In the second trial, the examined sections of fish stomach tunica mucosa and tunica submucosa showed normal histological patterns, without inflammations (lymphocyte infiltration) or degenerations in both CTRL and IM fish. Both the tunica mucosa and the tunica submucosa appeared regularly extended, without interruptions or alterations of the epithelium; and the cells were intact and did not show any sign of suffering (Figure 5). 

## 3. Discussion

The gradual increase in environmental eutrophication resulting from mariculture effluents represents a major issue imposing an urgent need to mitigate this negative impact to the marine ecosystem [24,25]. Bioremediation represents a valid solution, particularly in integrated multi-trophic aquaculture systems, where bioremediator organisms are employed providing the final self-purification [5], and transforming the wastes into useful biomass [2]. In the present study, we utilized an integrated bioremediation approach to realize an IMTA system involving polychaetes and macroalgae reared/cultivated in association with fish cages, achieving, for both organisms, consistent amounts of biomass, and opening several other interesting new horizons. In addition to the high production of bioremediators biomass, thanks to the realized IMTA system, a restoration of the aquaculture rearing environment was achieved in terms of microbial contamination (i.e., total coliforms and *Escherichia coli*) and nutrient concentrations (i.e., phosphorous and nitrogen salts) (L. Stabili unpublished data) as well as an amelioration of the benthic communities under the cages. (A. Giangrande unpublished data).

From the obtained results several interesting issues arose.

Both in the case of macroalgae and polychaetes, a consistent amount of biomass were achieved in the aquaculture plant. The algal species selected in the present study was cultivated in the multitrophic integrated aquaculture scenario to remove nitrogen and phosphorus surplus from waste, and at the same time to become a valuable by-product of bioremediation. This species already showed to be an effective bioremediator in integrated cultivation systems with fish and crustacean, since the nutrient concentration in the surrounding environment resulted considerably reduced [26,27]. In the here-realized IMTA system, the cultivation of *C. linum* resulted in a good biomass increase reaching about a 5% SGR. This result is noteworthy leading to suggest that this seaweed biomass could be employed as a potential source of nutritionally beneficial compounds for animal and human consumption. In order to exploit the obtained algal biomasses biochemical analyses on the total lipid and fatty acids content were performed revealing a concentration of lipids corresponding to 9.4 ± 2.4 mg/g DW with an interesting content of PUFAs. In particular, n-3 and n-6 fatty acids were mainly represented by linoleic, linolenic, docosahexaenoic (DHA), eicosapentaenoic acid (EPA), and arachidonic (ARA) acids. The presence of EPA and DHA is valuable since fish oil, used for the formulation of fish feeds, is the most common and major source of the n-3 fatty acids EPA and DHA, making the fish more beneficial for consumers. However, since the fish resource is decreasing whilst fish oil price is increasing as well as the commercial interest in these long chain fatty acids [28,29], there is an urgent need for an alternative source of essential PUFAs. In this regard, some farmers are opting to use cheaper alternatives such as vegetable oil, cotton seed oil, and sun-flower oil that; however, often result poor in terms of n-3 EPA and DHA. The commercial production of DHA and EPA from algae became a lively business in the last part of the twnetieth century due to the increase in awareness of their benefits for human health. In particular, DHA showed beneficial effects on preventing human cardiovascular diseases, cancer, schizophrenia, and Alzheimer’s disease [30]. Moreover, n-3 fatty acids, as well as arachidonic acid, are necessary in the growth and functional development of the brain and the maintenance of the normal brain function in adults. Mammals are unable to produce them and consequently they must be supplied as food supplement, considering that an insufficient fatty acid consumption is the major cause of human chronic diseases.

The experience of rearing polychaetes in IMTA also gave encouraging results showing that a huge biomass can be achieved. The slight mortality recorded, coupled with the high increase in volume and biomass of the reared polychaetes, demonstrated the feasibility of co-rearing *S. spallanzanii* with low costs of production and high profits. As a consequence, if the acquired technology is applied to a medium aquaculture farm, the produced polychaete biomass, coupled to algal and fish biomass, might reach values in the order of several tons/year in a completely non-fed culturing system. In addition, a reduction of the environmental impact due to aquaculture activity is accomplished on account of the well-known bioremediation capabilities of *S. spallanzanii* [7,31]. On the other hand, the capability of this polychaete to remove several bacterial groups from fish farm waste, and the bioremediation service associated to the waste conversion into polychaete protein-rich biomass of potentially marketable value, had been already stated by Stabili et al. [32]. From the present study it can be inferred that the use of the here-investigated invertebrate might ensure not only protein but also lipid and fatty acid supply for fish feeds. Obtained data indeed evidenced an interesting composition also in terms of fatty acids profile. In particular, long-chain polyunsaturated ω-3 fatty acids accounted for 5.6% of PUFAs and long-chain polyunsaturated n-6 fatty acids for 9.9%. In addition, the presence of the essential α-linolenic acid (18:3 n-3) in the investigated worm is not to be underrated, since up to now the main known sources for this fatty acid are fish and vegetable oils [33]. Moreover, World Health Organization’s recommendation indicate that n-6/n-3 ratio should not be higher than 10 in the human diet [34]. 

Even though the total lipid content is usually low in macroalgae, their employment as an ingredient in aquaculture feeds is well established on account of their presence of PUFAs, combined with other interesting secondary metabolites (e.g., polysaccharides, vitamins, proteins, antibiotics) [35,36,37]. In the present research, as new dietary ingredients, *C. linum* was carefully mixed with *S. spallanzanii* (10% of polychaetes and 5% of algae) for the formulation of an innovative feed for farmed juveniles of seabass *D. labrax*. The analysis of the productive indexes obtained from the growth trials showed that:

- During both the trials, the physico-chemical parameters of water were in the normal range for optimal fish growth, showing that the innovative feed did not affect negatively the rearing conditions;
- Employing the innovative meal, the survival rate was 96% ± 2% with the lowest sized fish and 94% ± 2%. with the highest sized fish. Being that these values higher were than 80%, our results can be considered excellent in nursery operation [38]. Thus, our data indicate that the innovative meal did not affect the survival of treated fish and that the fish rearing was carefully managed. Moreover, high values of survival rate indicate that all fish had equal access to feed and uniform growth rates were achieved as demonstrated by the obtained indices of weight and length increase;
- In both the trials no statistical differences were evidenced in biomass gain and specific growth rate between fishes nourished with the control and the innovative meal. These results are of particular interest since they demonstrated that the addition to fishmeal of 10% of polychaete meal and 5% of algal meal does not produce negative effects on the fish growth. This result is in line with other studies showing positive effects on growth, feed utilization, lipid metabolism, liver function, body composition, stress responses, and disease resistance determined by the addition of even small amounts of several algal-based meals to fish diets [39]. Moreover, the polychaete *S. spallanzanii*, due to its already investigated high protein content with noble amino acids [15] presumably contributed to improve palatability for the farmed fish species. This represents an added value in the hypothesis of fishmeal replacement, since decrease of fish meal, and/or fish oil, can lead to a decrease in palatability in diets with, as an example, an increased vegetal content. The decrease of palatability can lead to a decrease of feed uptake, making these feeds less effective for fish growth and health. Moreover, the experimental feed containing polychaete and seaweed meal did not produce any histological alterations in the stomach of fish and the conditions of stomach mucosa were comparable with those of fish fed with the control feed.

The here presented data, although preliminary, demonstrated the reliability and the non-toxicity of the innovative feed; however, further investigations employing higher percentages of *C. linum* and *S. spallanzanii* are needed in order to set the best fish meal recipe, providing fish with additional beneficial compounds and also partially replacing fishmeal.

## 4. Materials and Methods

### 4.1. Species Sampling

**Figure 6:**
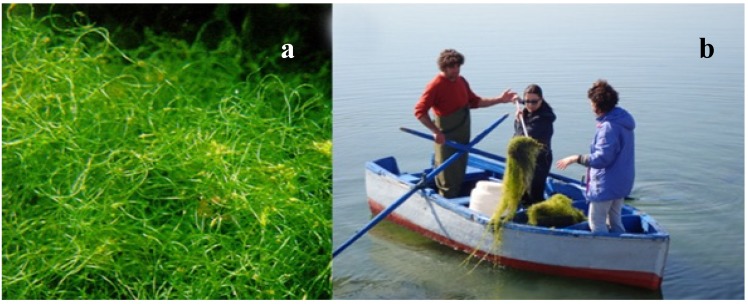
*Chaetomorpha linum* from the Mar Piccolo of Taranto: (**a**) Thalli; (**b**) hand-collection.

As regards macroalgae (Figure 6), the selected species was *Chaetomorpha linum* (Chlorophyta, Cladophorales) characterized by filamentous, uniseriate, unbranched thalli, of a pale bright green colour, 200–1000 μm wide and from 10 cm up to several meters long, commonly found both in the attached and in the unattached habitus (Figure 6a). Thalli of *C. linum* were hand-collected by means of a rake in the Mar Piccolo of Taranto (Mediterranean Sea, Ionian Sea, Italy) (Figure 6b), where it can make very thick drifting mattresses, in the period late autumn–late spring, with highly variable biomass values throughout the year.

Specimens of *S. spallanzanii* were obtained from the natural recruitment on plastic nets used as collectors placed in the fish farm located in the Gulf of Taranto (Mediterranean Sea, Ionian Sea, Italy) (Figure 3a) until they reached a mean initial biomass of 580 ± 150 mg.

Both macroalgae and worms were randomly divided in three sets The first set was placed around the fish cages in the realized IMTA system for bioremediation purposes, the second set was employed for the biochemical analyses, and the third set was used for the preparation of the innovative fish feed. 

### 4.2. Rearing/Cultivation of Bioremediators in IMTA System

In order to obtain high algal biomass, *C. linum* cultivation trials were realized with the first set of *C. linum*, in an IMTA system equipped with fish cages within the framework of the Remedia-Life Project (LIFE16 ENV/IT/000343). The experimental design of the project consisted of both the IMTA system including 6 fish cages, where the waste restoration was realized by the presence of the selected bioremediators and a control area where fishes were reared without bioremediators. Fish cages had a diameter of about 20 m (circumference of about 60 m) fixed to the bottom through appropriate buoys. 

The collected seaweeds were transferred to the aquaculture farm to set up the cultivation sockets, each consisting in seaweeds enclosed into a net sack and hung with a festoon arrangement at 1 m of depth within a mussel long-line system located around 6 fish cages (Figure 1 and Figure 2). A total of 252 cultivation sockets were allocated in the plant, hung for 6 months until seaweed reached the highest biomass, after that the surplus was collected. Later, since thalli fragmentation was observed, followed by a rapid decay, cultivation was stopped. Seaweed biomass growth was measured according to the standard formulation of specific growth rate (SGR) for seaweeds as [40]:

[(Wt/W0)1/t − 1] × 100%.

Polychaetes employed for the bioremediation purpose, as already specified, were obtained from the natural recruitment on plastic nets used as collectors immersed in the fish farm (Figure 3). A total of 252 collectors were placed around the fish cages suspended at about 12 m depth. The worm biomass was measured after 3 and 6 months of permanence in the realized IMTA system.

### 4.3. Total Lipid and Fatty Acid Analysis

The second set of macroalgae and polychaetes was transported to the laboratory under refrigeration. Here, macroalgal thalli were placed into suitable tanks, cleaned of possible epibionts and detritus, rinsed with seawater and then with an 8‰ sterile physiological solution. Macroalgae were then placed in a stove at a temperature of 50 °C for 24 h and subsequently were ground with the help of a mortar and a pestle. All the analyses were done with ground algal tissues. Afterwards, biochemical analyses including total lipids and fatty acids determination were carried out.

Animals were rinsed with seawater filtered with 0.45 µm Millipore filters to remove any possible epibionts and then extracted from their tubes, dried at 60 °C, minced with a blender (Kinematica Type MB 550), and then employed for the biochemical analyses including total lipids and fatty acids evaluation. 

All reagents and solvents (analytical grade) were acquired from Sigma (Sigma–Aldrich GmbH, Steinheim, Germany). Total lipids from macroalgae and polychaetes were extracted in accordance with the method described in Folch et al. [41]. All the samples (dry macroalgal tissues or polychaete homogenized tissues) were extracted with methanol:chloroform:water (1:2:1) in order to obtain a final volume 20 times the sample volume. Lipids were obtained after centrifugation and removal of the upper phase and collection of the lower chloroform phase. The evaluation of the total lipid content was obtained by a colorimetric enzymatic assay [42] employing a commercial kit (FAR, Verona, Italy). 

The fatty acids composition of macroalgae and polychaetes was determined in accordance with the method described in Budge and Parrish [43]. Briefly, the fatty acids (FAs) of total lipids were transesterified to methyl esters as described by Stabili et al. [13,44]. The samples were cooled, and, after the addition of 1 mL of distilled water, shaken vigorously. Fatty acid methyl esters (FAMEs) were collected in the upper benzene phase. Benzene phase was moved to a vial and subjected to dry conditions by using a nitrogen stream at a very slow applied flow rate to avoid the loss of the sample. Gas chromatography using an HP 6890 series GC (Hewlett Packard, Wilmington, DE, USA) equipped with flame ionization detector was employed to perform the analyses of sample FAME extracts. In order to separate the FAMEs an Omegawax 250 capillary column (Supelco, Bellafonte, PA, USA) (30 mm long, 0.25 mm internal diameter, and 0.25 mm film thickness) was utilized. The column temperature program was the following: 150–250 °C at 4 °C/min. and then held at 250 °C. The retention times of known standards (FAME mix, Supelco-USA) were used to attain the right FAMEs identification The results were indicated as percentages of total identified methyl ester fatty acids. The employed gas carrier was helium at a flow of 1 mL/min. The injected volume was 1 μL. 

All assays were conducted in replicate samples of the macroalgae and worms. 

### 4.4. Experimental Feed Fish Formulation and Proximate Composition

The third set of macroalgae and polychaetes was placed on aluminum foils and dried in oven at 60 °C for 48 h. Dried material was then ground to obtain algal (AS) and polychaete (PS) powder to be used as ingredient to prepare the experimental fish feed. For this purpose, oil and dry ingredients were thoroughly mixed, whereby water was then blended into the mixture to attain an appropriate consistency for pelleting using a meat grinder. Pellets were dried overnight at 50 °C and refrigerated at 6 °C until utilization. Two feeds were prepared: A control fishmeal-based feed (CTRL) and an innovative feed (innovative meal = IM) containing 10% polychaete (PS) and 5% algae (AS). The composition of the two feeds is reported in Table 2. 

The prepared feeds (CTRL and IM) were also analyzed (*n* = 3) to determine their proximate composition in accordance with standard methods [45]. 

The gross energy (GE) content was determined by an adiabatic calorimetric bomb (IKA C7000, Staufen, Germany). Total nitrogen content was determined in accordance with the Dumas method, using a nitrogen analyzer (Rapid N III, Elementar Analysensysteme GmbH, Hanau, Germany). The crude protein was calculated as total N × 6.25. 

### 4.5. Fish Growth Trials

The innovative feed as well as the control feed were utilized for feeding juveniles of European sea bass *Dicentrarchus labrax*. The study was carried out between May 2019 and September 2019 for 4 months in experimental tanks at the fish farm. In a first trial, juveniles of *D. labrax* at 36 days of age with an initial mean body weight of 0.05 ± 0.01 g were randomly stocked (200 fish for tank) in 6 fiberglass tanks (80 L) supplied by an open water circuit: Three tanks for the control feed (CTRL) and three tanks for the innovative feed (IM). The first trial lasted 60 days from May 2019 to July 2019. Fish were singularly weighted by an Analytical Balance Cubis^®^ MSA SARTORIUS (readability 0.01 mg) (*n* = 34 for each tank), after 30, 45, and 60 days, in order to check the fish biomass gain. The fish were fed to satiation by hand twice a day, 7 days per week. Temperature and dissolved oxygen were determined daily in the morning and in the afternoon with a digital oximeter (YSI 55 Hexis). 

The same procedures described above were also utilized in a second trial, which lasted 30 days from August 2019 to September 2019. In this case, *D. labrax* juveniles at 153 days of age with an initial mean body fish weight of 2.00 ± 0.47 g were employed.

At the end of both experimentation trials, survival rate (%), biomass growth, specific growth rate, and coefficient of variation for length were evaluated with the following formulas [46]:

Survival rate (%) = (number of fish at the end/number of fish at the beginning) × 100;
Biomass gain (g) = final individual weight − initial individual weight;
Specific growth rate (%) = (ln final weight − ln initial weight) × 100/feeding days.
Feed intake (g kg^−1^day^−1^) = feed consumed/biomass/t.

### 4.6. Fish Histological Analyses

In order to control the histology of the stomach tunica mucosa and tunica submucosa, six fish for each experimental tank (CTRL and IM) were sampled at the beginning and at the end of the second trial. The stomachs were excised from fish and samples were fixed in buffered formalin in order to assess eventual alterations induced by the experimental diet. After dehydration and embedding in paraffin wax following standard histological techniques, the histological samples were cut (5 μm thickness) by a microtome and stained with hematoxylin and eosin after 24 h in a laboratory thermostat. All the animal experiments were approved by the Bioethical Committee of the University of Turin (Protocol Number 47528). All efforts were made to minimize the animals’ suffering.

### 4.7. Statistical Analysis

Analysis of variance (ANOVA)-1-way was used to test for differences in the weight, length, survival rate, biomass gain, and specific growth rate between fish fed with the innovative feed and fish fed with the control feed. All the analyses were performed by using GMAV 5 computer program (University of Sidney, Australia).

## 5. Conclusions

The high biomass of *Sabella spallanzanii* and *Chaetomorpha linum*, obtained as by-product of the bioremediation process in the realized IMTA system, leads to suggest their employment as sources of functional ingredients beneficial to fish and human health. On the basis of our results from the experimental feeding trials on *Dicentrarchus labrax* juveniles, we can conclude that the prepared innovative feed containing polychaetes and algae as ingredients represents a first step in the optimization of an innovative meal for the European seabass practical diets. The employment of the examined species as a dietary ingredient in fish feed could contribute to overcome the major concerns over fish as a declining resource and the consequent rising cost of fish feeds worldwide. Work is ongoing to explore some of these issues in detail. 

## Figures and Tables

**Table 1:** Fatty acid composition of *Sabella spallanzanii*.

**Saturated fatty acid (SFA) percentages**	
14:0	9.64 ± 0.57
15:0	0.25 ± 0.02
16:0	26.18 ± 3.82
17:0	0.20 ± 0.01
18:0	8.95 ± 0.32
20:0	1.66 ± 0.01
22:0	7.62 ± 0.21
23:0	0.34 ± 0.09
24:0	2.43 ± 0.86
∑	57.26
**Monounsaturated fatty acid (MUFA) percentages**	
16:1 n-7	5.62 ± 1.36
17:1 n-8	4.67 ± 1.62
18:1 n-9	3.16 ± 0.62
18:1 n-7	4.94 ± 0.98
20:1 n-9	3.28 ± 1.15
22:1 n-9	1.12 ± 0.06
24:1 n-9	3.67 ± 0.13
∑	26.46
**Polyunsaturated fatty acid (PUFA) percentages**	
18:2 n-6	0.54 ± 0.08
18:2 n-4	0.64 ± 0.10
18:3 n-6	0.81 ± 0.16
18:3 n-3	2.04 ± 1.18
20:2 n-6	0.85 ± 0.24
20:3 n-6	1.49 ± 0.13
20:3 n-3	1.20 ± 0.23
20:4 n-6	1.74 ± 0.39
20:5 n-3	1.17 ± 0.32
22:2 n-6	4.50 ± 1.25
22:6 n-3	1.29 ± 0.36
∑	16.28

**Table 2:** Composition of the experimental diets.

Ingredients (%)	^1^ CTRL	^1^ IM
Fish meal ^2^	88.0	73.0
Algae meal	-	5.0
Polychaete meal	-	10.0
Fish oil ^2^	9.0	9.0
Lygnumsulphyte	1.0	1.0
Mineral mixture ^3^	1.0	1.0
Vitamin mixture ^4^	1.0	1.0

^1^ CTRL: Control feed; IM: Innovative feed. ^2^ Bioceval GmbH and Co. KG, Germany. ^3^ Mineral mixture (mg/kg diet): bicalcium phosphate, 500 g; calcium carbonate, 215 g; sodium salt, 40 g; potassium chloride, 90 g; magnesium chloride, 124 g; magnesium carbonate, 124 g; iron sulfate, 20 g; zinc sulfate, 4 g; copper sulfate, 3 g; potassium iodide, 4 mg; cobalt sulfate, 20 mg; manganese sulfate, 3 g; sodium fluoride, 1 g (Granda Zootecnica, Cuneo, Italy).^4^ Vitamin mixture (IU or mg/kg diet): dl-a-tocopherol acetate, 60 IU; sodium menadione bisulfate, 5 mg; retinyl acetate, 15,000 IU; cholecalciferol, 3000 IU; thiamin, 15 mg; riboflavin, 30 mg; pyridoxine, 15 mg; B12, 0.05 mg; nicotinic acid, 175 mg; folic acid, 500 mg; inositol, 1000 mg; biotin, 2.5 mg; calcium pantothenate, 50 mg; choline chloride, 2000 mg (Granda Zootecnica, Cuneo, Italy).

**Table 3:** Proximate composition (% dry weight) of the experimental diets (*n* = 3). Values are reported as mean ± S.E.

(% Dry Weight)	^1^ CTRL	^1^ IM
Crude protein	46.0 ± 0.4	45.8 ± 0.3
Ether extract	15.5 ± 0.1	15.3 ± 0.1
Ash	11.5 ± 0.1	11.2 ± 0.2
Gross energy	20.79 ± 0.21	20.82 ± 1.01

^1^ CTRL: Control feed; IM: Innovative feed.

**Table 4:** Survival rate, biomass growth, specific growth rate, final weight, and feed intake of *Dicentrarchus labrax* fed with the experimental feeds (each value represents the mean ± S.D.). The initial mean fish weight was 0.05 ± 0.01 g for the I Trial and 2.00 ± 0.47 for the II Trial.

**I Trial**		
	**^1^ CTRL**	**^1^ IM**
Biomass gain (g)	0.62 ± 0.04	0.55 ± 0.01
Specific growth rate (%)	4.32 ± 0.21	4.14 ± 0.16
Survival rate (%)	87 ± 2	96 ± 2
Final fish weight (g)	0.67 ± 0.05	0.6 ± 0.07
Feed intake (g kg^−1^day^−1^)	15.0 ± 1.3	14.2 ± 1.6
**II Trial**		
	**^1^ CTRL**	**^1^ IM**
Biomass gain (g)	2.87 ± 0.15	2.1 ± 0.12
Specific growth rate (%)	2.96 ± 0.16	2.4 ± 0.11
Survival rate (%)	85 ± 3	94 ± 2
Final fish weight (g)	4.87 ± 0.8	4.1 ± 0.9
Feed intake (g kg^−1^day^−1^)	15.5 ± 0.4	14.7 ± 1.3

^1^ CTRL: Control feed; IM: Innovative feed.

## References

[1] FAO (2018). The State of World Fisheries and Aquaculture 2018—Meeting the Sustainable Development Goals.

[2] Chopin T., Christou P., Savin R., Costa-Pierce B.A., Misztal I., Whitelaw C.B.A. (2013). Aquaculture, Integrated Multi-trophic (IMTA). Sustainable Food Production.

[3] Gifford S., Dunstan R.H., O’Connor W., Koller C.E., MacFarlane G.R. (2006). Aquatic zooremediation: Deploying animals to remediate contaminated aquatic environments. Trends Biotechnol..

[4] Riisgård H.U., Larsen P.S. (1995). Filter-feeding in marine macro-invertebrates: Pump characteristics, modelling and energy cost. Biol. Rev..

[5] Ostroumov S. (2005). Some aspects of water filtering activity of filter feeders. Hydrobiologia.

[6] Milanese M., Chelossi E., Manconi R., Sara A., Sidri M., Pronzato R. (2003). The marine sponge *Chondrilla nucula* Schmidt, 1862 as an elective candidate for bioremediation in integrated aquaculture. Biomol. Eng..

[7] Giangrande A., Cavallo A., Licciano M., Mola E., Pierri C., Trianni L. (2005). Utilization of the filter feeder *Sabella spallanzanii* as bioremediator in aquaculture. Aquac. Int..

[8] Fu W., Sun L., Zhang X., Zhang W. (2006). Potential of the marine sponge *Hymeniacidon perleve* as a bioremediator of pathogenic bacteria in integrated aquaculture ecosystems. Biotechnol. Bioeng..

[9] Ostroumov S., Widdows J. (2006). Inhibition of mussel suspension feeding by surfactants of three classes. Hydrobiologia.

[10] Ajjabi L.C., Abaab M., Segni R. (2018). The red macroalga *Gracilaria verrucosa* in co-culture with the Mediterranean mussels *Mytilus galloprovincialis*: Productivity and nutrient removal performance. Aquacult Int.

[11] Martins A., Vieira H., Gaspar H., Santos S. (2014). Marketed marine natural products in the pharmaceutical and cosmeceutical industries: Tips for success. Mar. Drugs.

[12] Duarte K., Justino C.I.L., Pereira R., Freitas A.C., Gomes A.M., Duarte A.C., Rocha-Santos T.A.P. (2014). Green analytical methodologies for the discovery of bioactive compounds from marine sources. Trends Environ. Anal. Chem..

[13] Stabili L., Acquaviva M.I., Biandolino F., Cavallo R.A., De Pascali S.A., Fanizzi F.P., Narracci M., Petrocelli A., Cecere E. (2012). The lipidic extract of the seaweed *Gracilariopsis longissima* (Rhodophyta, Gracilariales): A potential resource for biotechnological purposes?. New Biotechnol..

[14] Li P., Mai K., Trushenski J., Wu G. (2009). New developments in fish amino acid nutrition: Towards functional and environmentally oriented aquafeeds. Amino acids.

[15] Stabili L., Sicuro B., Daprà F., Gai F., Abete C., Dibenedetto A., Pastore C., Schirosi R., Giangrande A. (2013). The Biochemistry of *Sabella spallanzanii* (Annelida: Polychaeta): A potential resource for the fish feed industry. J. World Aquac.Soc..

[16] Salze G., Mc Lean E., Battle P.R., Schwarz M.C., Craig S.R. (2010). Use of soy protein concentrate and novel ingredients in the total elimination of fish meal and fish oil in diets for juvenile cobia, *Rachycentron canadum*. Aquaculture.

[17] Olive P.J.W., Craig S., Cowin P.B.D., Islam M.D., Rutherford G. (2002). The culture of Polychaeta as a contribution to sustainable production of aquafeeds. Abstract of Aquaculture Europe 2001.

[18] Murugesan P., Elayaraja S., Vijayalakshmi S., Balasubramanian T. (2011). Polychaetes—A suitable live feed for growth and colour quality of the clownfish, *Amphiprion sebae* (Bleeker, 1953). J. Mar. Biol. Assoc. India.

[19] García-Alonso J., Müller C.T., Hardege J.D. (2008). Influence of food regimes and seasonality on fatty acid composition in the ragworm. Aquat. Biol..

[20] Mustafa M.G., Nakagawa H. (1995). A review: Dietary benefits of algae as an additive in fish feed. Isr. J. Aquac..

[21] Valente L.M.P., Gouveia A., Rema P., Matos J., Gomes E.F., Pinto I.S. (2006). Evaluation of three seaweeds *Gracilaria bursa-pastoris*, *Ulva rigida* and *Gracilaria cornea* as dietary ingredients in European sea bass (*Dicentrarchus labrax*) juveniles. Aquaculture.

[22] Yu Y.Y., Chen W.D., Liu Y.J., Niu J., Chen M., Tian L.X. (2016). Effect of different dietary levels of *Gracilaria lemaneiformis* dry power on growth performance, haematological parameters and intestinal structure of juvenile Pacific white shrimp (*Litopenaeus vannamei*). Aquaculture.

[23] Miller M.R., Nichols P.D., Carter C.G. (2008). n-3 Oil sources for use in aquaculture—alternatives to the unsustainable harvest of wild fish. Nutr. Res. Rev..

[24] Martinez-Porchas M., Martinez-Cordova L.R. (2012). World Aquaculture: Environmental Impacts and Troubleshooting Alternatives. Sci. World J..

[25] Ahmed N., Thompson S. (2019). The blue dimensions of aquaculture: A global synthesis. Sci. Total Environ..

[26] Bouwman A.F., Pawlowski M., Liu C., Beusen A.H.W., Shumway S.E., Glibert P.M., Overbeek C.C. (2011). Global hindcasts and future projections of coastal nitrogen and phosphorus loads due to shellfish and seaweed aquaculture. Rev. Fish. Sci..

[27] Chopin T., Buschmann A.H., Halling C., Troell M., Kautsky N., Neori A., Kraemer G.P., Zertuche-González J.A., Yarish C., Neefus C. (2001). Integrating seaweeds into marine aquaculture systems: A key toward sustainability. J. Phycol..

[28] Su J., Hou H., Wang C., Luo Y. (2019). Effects of replacing soybean meal with cottonseed meal on growth, muscle amino acids, and hematology of juvenile common carp, *Cyprinus carpio*. Aquac. Int..

[29] Linder M., Belhaj N., Sautot P., Tehrany E.A. (2010). From Krill to Whale: An overview of marine fatty acids and lipid compositions. Oléagineux Corps Gras Lipides.

[30] Ibarguren M., López D.J., Escribá P.V. (2014). The effect of natural and synthetic fatty acids on membrane structure, microdomain organization, cellular functions and human health. Biochim. Biophys. Acta Biomembr..

[31] Stabili L., Licciano M., Giangrande A., Fanelli G., Cavallo R.A. (2006). *Sabella spallanzanii* filter-feeding on bacterial community: Ecological implications and applications. Mar. Environ. Res..

[32] Stabili L., Schirosi R., Licciano M., Mola E., Giangrande A. (2010). Bioremediation of bacteria in aquaculture waste using the by-product polychaete *Sabella spallanzanii*. New Biotechnol..

[33] Sanchez-Machado D.I., Lopez-Cervantes J., Lopez-Hernandez J., Paseiro-Losada P. (2004). Fatty acids, total lipid, protein and ash contents of processed edible seaweeds. Food Chem..

[34] Mahan L.K., Escott-Stump S. (2000). Krause’s Food, Nutrition, & Diet Therapy.

[35] Colombo M.L., Risè P., Giavarini F., De Angelis L., Galli C., Bolis C.L. (2006). Marine macroalgae as sources of polyunsaturated fatty acids. Plant Food Hum. Nutr..

[36] Dawczynski C., Schubert R., Jahreis G. (2007). Amino acids, fatty acids, and dietary fibre in edible seaweed products. Food Chem..

[37] Cecere E., Acquaviva M., Belmonte M., Biandolino F., Cavallo R.A., Lo Noce R., Narracci M., Petrocelli A., Ricci P., Stabili L. (2010). Seaweeds and aquaculture: An indispensable alliance for the integrated management of coastal zone. Biol. Mar. Mediterr..

[38] Sumi K.R., Das M., Siddika I. (2011). Effect of different protein levels of fry feed on the production of quality tilapia (*Oreochromis niloticus*) fry. J. Bangladesh Agric. Univ..

[39] Shi Q., Rong H., Hao M., Zhu D., Aweya J.J., Li S., Wen X. (2019). Effects of dietary *Sargassum horneri* on growth performance, serum biochemical parameters, hepatic antioxidant status, and immune responses of juvenile black sea bream *Acanthopagrus schlegelii*. J. Appl Phycol..

[40] Yong Y.Y., Yong W.T.L., Anton A. (2013). Analysis of formulae for determination of seaweed growth rate. J. Appl. Phycol..

[41] Folch J., Less M., Stanley G.H.S. (1957). A simple method for the isolation and purification of total lipids from animal tissues. J. Biol. Chem..

[42] Zollner N., Kirsch K. (1962). Determination of the total lipid concentration in serum. Zentralblatt Gesamte Exp. Med..

[43] Budge S.M., Parrish C.C. (2003). FA determination in cold water marine samples. Lipids.

[44] Stabili L., Acquaviva M.I., Biandolino F., Cavallo R.A., De Pascali S.A., Fanizzi F., Narracci M., Cecere E., Petrocelli A. (2014). Biotechnological potential of the seaweed *Cladophora rupestris* (Chlorophyta, Cladophorales) lipidic extract. New Biotechnol..

[45] AOAC (1995). Official Methods of Analysis.

[46] Palmegiano G.B., Daprà F., Forneris G., Gai F., Gasco L., Guo K., Peiretti P.G., Sicuro B., Zoccarato I. (2006). Rice protein concentrate meal as a potential ingredient in practical diets for rainbow trout (*Oncorhynchus mykiss*). Aquaculture.

